# Antibiotic resistance gene analyses in microbial communities: challenges and opportunities

**DOI:** 10.1038/s41467-026-71462-4

**Published:** 2026-04-04

**Authors:** D. G. Joakim Larsson, Carl-Fredrik Flach, Erik Kristiansson

**Affiliations:** 1 Department of Infectious Diseases, Institute of Biomedicine, University of Gothenburg, Gothenburg, Sweden; 2 Centre for Antibiotic Resistance Research in Gothenburg (CARe), University of Gothenburg, Gothenburg, Sweden; 3 Department of Mathematical Sciences, Chalmers University of Technology and University of Gothenburg, Gothenburg, Sweden

**Keywords:** Antimicrobial resistance, Metagenomics, Bacterial infection

## Abstract

There is an increasing interest in studying antibiotic resistance genes in microbial communities, however, there is no unified way to identify them in metagenomics datasets or to interpret the risks associated with them. In this Comment, the authors discuss current technical challenges and how to mitigate them.

## Teaser

Culture-independent antibiotic resistance gene analyses enable broad explorations of microbial communities but often fail to link such genes to bacterial hosts and genetic contexts. This makes assessing prevalence of resistant pathogens and likelihood of further transmission or resistance evolution uncertain.

## Rationale for studying antibiotic resistance genes

Antibiotic resistance has become one of the most pressing global health challenges to date. As bacteria and their genetic material tend to move between humans, domestic animals and external environments, there is a need for both interventions and research across the entire One-Health spectrum. Dynamics within microbial communities are complex: Non-pathogenic bacteria may act as sources or intermediary carriers of genetic resistance determinants or can, without being resistant themselves, impact the success of resistant bacteria in the same community. Given the challenges in cultivating the majority of bacterial species, culture-independent analyses such as metagenomic sequencing or polymerase chain reactions (PCR) have provided opportunities to gain a more holistic view of antibiotic resistance genes (ARGs) in microbial communities, far beyond individual cultivable pathogens. Consequently, studies of the nature and abundance of ARGs are today used as the basis for addressing intriguing questions. These include quantifying transmission risks and routes of resistant pathogens, understanding selection pressures on microbial communities for resistance, and providing insights into regional resistance situations^1^.

## Analysing ARGs in communities—technical limitations and solutions

For decades, scientists have analysed ARGs via (quantitative) PCR in complex samples such as waste waters, soils and human microbial communities. PCR sensitively measures individual abundances of genes, and high-throughput PCR arrays or multiplexing approaches^2,3^ can analyse hundreds of ARGs in parallel; however, given the millions of predicted and identified ARGs^4^, a priori defined PCR arrays may overlook many relevant ones. PCR is, furthermore, inherently sensitive to non-specific primer binding, leading to high risks for false positives and erroneous quantification. This risk often becomes evident when working with highly diverse microbial environmental samples, such as waste waters that contain many similar, potentially cross-reacting gene sequences, calling for better validation under realistic conditions.

High-throughput sequencing allows for a random, broad and deep shotgun approach that can essentially identify any ARG, thereby circumventing the challenge of non-specific PCR primer binding. Such sequencing technologies also paved the way for studying any gene recognisable as an ARG, as long as a similar gene is present in a reference database. As the choice of which ARGs to look for can be made after the data are generated, the sequencing data can be reused for retrospective ARG analyses. In addition, the same data enable studies of taxonomic composition and other biochemical functions. Despite improved accuracy of newer sequencing technologies, insufficient sequencing depth, which refers to the total amount of DNA sequenced, remains a limitation in many applications given the high diversity of most microbial communities. Hence, a major remaining challenge with shotgun metagenomics is to detect and quantify anything but the most commonly occurring ARGs.

**Fig. 1:**
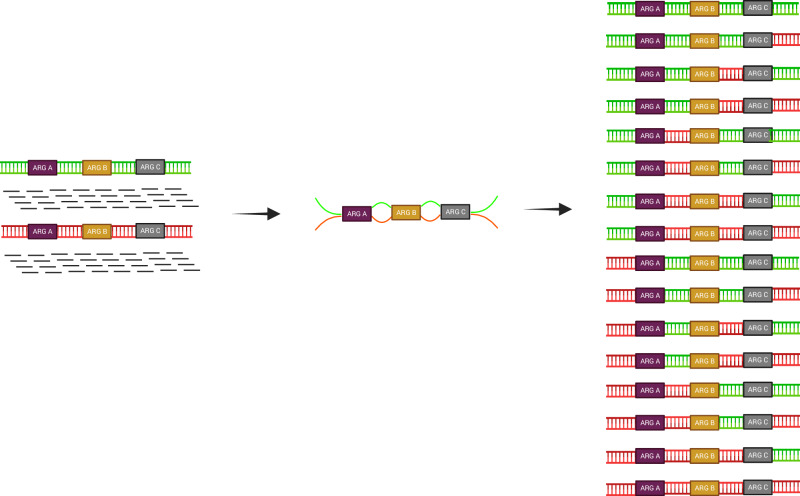
There are many tools that will provide assemblies of short DNA reads from shotgun metagenomes. A challenge is that the reads (or paired reads) do not span across both sides of highly mobile sequences (such as ARGs) occurring in multiple species, strains and contexts. The figure illustrates the conceptual challenge where short-read sequences of DNA (black lines, left side of figure) from two bacteria with different genetic backgrounds (red, green) carrying single copies of three identical ARGs (purple, brown and grey boxes) can be assembled (middle) in 16 different ways (right). Fourteen of these are artefacts. In metagenomes, high complexity and limited coverage makes assemblies much less accurate than for genomes^5^. As ARGs are often located on plasmids, additional approaches are typically needed to assign ARGs to hosts. Created in BioRender. Larsson, J. (2026) https://BioRender.com/axxb3vq.

Another critical limitation, shared with PCR, is placing ARGs in accurate genetic contexts. While there are numerous bioinformatic tools that assemble shorter DNA sequences from sequenced communities into longer, ARG-containing contigs, they often perform poorly when encountering genes or DNA sequences that are mobile and tend to occur in multiple contexts in different bacteria, which includes ARGs^5^. The underlying problem arises as the read (or read pair) does not typically span both sides of a mobile sequence. The assembly process will therefore often generate complex assembly graphs with multiple sequences both up- and downstream of every mobile sequence, with very limited possibilities, despite taking coverage into account, to conclude with certainty which ones are truly connected (Fig. 1). Risks for incorrect assemblies will increase as the number of mobile elements and the complexity of the community grow. Long-read sequencing, such as Oxford Nanopore and PacBio, has the potential to markedly reduce this problem. Nonetheless, benchmarking studies show that downstream analytical steps, particularly assembly and post-assembly processing, can represent a major source of artefacts even when using high-accuracy long reads, leading to chimeras, unsupported sequences, or misrepresented genomic features^6^. With parallelised technology platforms, some of the price paid in loss of sequencing depth compared to Illumina may also be regained^7^. A notable remaining difference is also the higher biomass typically required for long-read sequencing, which sometimes is a limiting factor.

Regardless of read length, regular sequencing will not connect plasmids with chromosomes. As the majority of clinically relevant ARGs are plasmid-borne and thus occur in multiple strains and species, it is often critical to link ARGs to species, or even to strains. A match of an assembled resistance plasmid to a plasmid previously reported in a given species does not mean it is hosted by that same species in the sequenced community. To resolve this challenge, the two most common approaches are Epic-PCR^8^ and Hi-C^9^, both of which link pieces of DNA that originate from within the same cell and are then sequenced together. However, in complex and dynamic microbial communities, Hi-C–based linkages can be difficult to interpret, as highly abundant taxa or multi-copy plasmids may generate spurious associations, particularly in systems with rapid population turnover or high viral predation. Sensitivity and resolution down to species and strains are still major challenges with both techniques, although long-read Epic-PCR and more accurate binning of Hi-C data might improve resolution to some extent.

An additional context-related challenge is the presence of extracellular DNA in community samples^10^. Associated risks for further transmission are apparently much smaller with free DNA as ARGs require successful transformation and incorporation into the genome of a new host to allow propagation, but how much smaller is unclear. Physical separation steps prior to sequencing are required to remove or separately analyse such DNA from genetic material present in live cells.

In its simplest form, ARGs are identified by matching DNA-reads against databases with known or predicted resistance genes, commonly CARD, Resfinder or ARGs-OAP. It is critical to carefully consider the content of the database, which may include not only mobile ARGs but also non-mobile ARGs, chromosomal resistance mutations, or resistance genes to antimicrobials other than antibiotics. Public metagenomic data are accumulating rapidly, providing a vast and important resource for the scientific community. However, the availability and quality of the associated metadata often limit their usefulness. Furthermore, similar to the skewness of bacterial species in genome databases, there is a strong overrepresentation of metagenomes from a limited set of environment types, in particular from humans, common domestic animals, waste waters and soils.

Only a small fraction of the ARGs in sequence repositories have been empirically shown to provide a resistance phenotype, and many are still to be discovered^4^. Exploring unknown ARGs is particularly valuable for newly developed antibiotics^11^. There are two common, principally different approaches to finding previously undescribed resistance genes in communities. Functional metagenomics based on the expression of random DNA fragments in a bacterial host allows identifying ARGs through screening transformants with antibiotics^12^. The advantage is that it does not rely on sequence similarities to known genes, but requires functionality in the heterologous host and has limited throughput. Predictive models from available genome and metagenome data have considerably higher throughput. Both Hidden Markov models^4^ which detect conserved sequence motifs, and, more recently, deep learning models^13^ which automatically extract informative patterns from genomic data, have proven useful. Ultimately, experimental validation remains essential to confirm the resistance phenotype, avoid overinterpretation of sequence-based matches, and to meaningfully assess the risks associated with putative ARGs.

## Interpretation of ARG data

Interpreting ARG data in metagenomes is associated with both technical and more conceptual/biological challenges. While long-read sequencing has historically been more error-prone than short-reads, accuracy is rapidly improving. In the context of ARG analyses, too relaxed cut-offs applied when matching sequences to those in ARG databases may lead to incorrect assignment of reads from close homologues that lack resistance functionality. Conversely, too stringent cut-offs may overlook clinically important ARG variants completely. Hence, cutoffs need to be carefully aligned with the specific genes, datasets and underlying question in mind. Another limitation is that relevant ARGs may be missing from the used database^4^, again leading to underestimations of ARGs. Normalisation of gene abundances to a reference value, such as the total reads or bacterial content, is critical, but which strategy is most appropriate depends on the questions asked^14^. Typically, ARG data are zero-inflated which some statistical methods deal with inappropriately, leading to considerable reductions in power^15^.

Still, we argue that the most concerning challenge lies in the biological interpretation. Increases in relative ARG abundances in communities are often interpreted as evidence of resistance selection. However, as ARGs are not equally distributed across species, any taxonomic change can lead to changes in ARG abundance that are entirely unrelated to resistance selection. Similarly, without reliable assignment to hosts, increased ARG abundance cannot simply be translated to increased transmission risks of relevant resistant pathogens. Furthermore, point mutations are in many cases highly important resistance determinants, but they are considerably more challenging to detect and quantify accurately in metagenomic data than mobile ARGs. Often, the definition of “antimicrobial resistance risks” or “AMR-risks” is lacking, which affects further downstream understanding of risks for transmission and different evolutionary processes. As drivers are likely to differ, clarity regarding the type of risks is critical for directing potential mitigation.

Risks related to simple bacterial transmission and to the evolution/emergence of novel resistance genotypes highly depend on context, most importantly the bacterial host species or even strain and, for resistance evolution, the immediate genetic context. An ARG located in a non-virulent strain is associated with vastly lower risk than the same ARG in a pathogen. Clinically important ARGs are often located on mobile plasmids, which create additional challenges for interpretation. Rapid transmission of resistance plasmids across species often occurs under the selection pressure from antibiotics. This means that not only the current bacterial host matters for risk assessment but also the potential host-range of the ARG-carrying plasmid, as well as the nature of other non-resistant compatible hosts present in the community. To understand risks, a more holistic assessment of the ecology of microbial communities is therefore needed, extending far beyond naïve gene-counting exercises. Models based on ARG abundances to assess transmission risk or resistance prevalence are often simple to generate. However, given the prevailing uncertainties around host, genetic context and transmission opportunities, risk-ranking schemes based on ARG rather than cultivation-based data currently stand on shaky grounds. A perhaps even more profound limitation with such approaches for risk assessment is that their validation against independently generated empirical data on health risks is a truly challenging endeavour and thus currently lacking. Without better context and validation, we should therefore interpret risks inferred from metagenomic ARG data with caution and refrain from quantitative assessments. It may be tempting to restrict interpretation to “relative risks”, but a reasonable estimate of absolute risks is also needed to avoid exaggerating health implications. We should also be humble and acknowledge that even with high-quality cultivation-based data at hand, it is often challenging to translate bacterial abundance in a given environment to infection risks.

## Conclusions and outlook

Next-generation sequencing and ARG analyses in communities have increased our understanding of resistance evolution and dynamics and will continue to do so in the years to come. As more genomes become available, including from those bacteria that are rare or difficult to cultivate, we will be better positioned to correctly interpret metagenomic data. Protocols that reach beyond long-read sequencing, including single-cell metagenomics, could become a future game-changer if further developed. Still, given current technologies, we need to appreciate the limitations of metagenomics and ARG analyses, particularly in relation to host, genetic contexts and the many challenges to infer health risks. It is also imperative that such limitations are reflected in policy initiatives that may be informed by ARG analyses^16^.

## References

[1] Larsson, D. G. J. & Flach, C.-F. Antibiotic resistance in the environment. *Nat. Rev. Microbiol.* **20**, 257–269 (2022). 34737424 10.1038/s41579-021-00649-x PMC8567979

[2] Ouyang, B. et al. Recent advances in environmental antibiotic resistance genes detection and research focus: from genes to ecosystems. *Environ. Int.* **191**, 108989 (2024). 39241334 10.1016/j.envint.2024.108989

[3] Knight, M. E. et al. National-scale antimicrobial resistance surveillance in wastewater: a comparative analysis of HT qPCR and metagenomic approaches. *Water Res.* **262**, 121989 (2024). 39018584 10.1016/j.watres.2024.121989

[4] Inda-Díaz, J. S. et al. Latent antibiotic resistance genes are abundant, diverse, and mobile in human, animal, and environmental microbiomes. *Microbiome* **11**, 44 (2023). 36882798 10.1186/s40168-023-01479-0 PMC9993715

[5] Abramova, A., Karkman, A. & Bengtsson-Palme, J. Metagenomic assemblies tend to break around antibiotic resistance genes. *BMC Genom.* **25**, 959 (2024). 10.1186/s12864-024-10876-0 PMC11479545 39402510

[6] Trigodet, F., Sachdeva, R., Banfield, J. F. & Eren, A. M. Troubleshooting common errors in assemblies of long-read metagenomes. *Nat. Biotechnol.*10.1038/s41587-025-02971-8 (2026). 41482538 10.1038/s41587-025-02971-8

[7] Mao, X. et al. Longitudinal metagenomic analysis on antibiotic resistome, mobilome, and microbiome of river ecosystems in a sub-tropical metropolitan city. *Water Res.* **274**, 123102 (2025). 39798533 10.1016/j.watres.2025.123102

[8] Liu, S. et al. Long-read epicPCR enhances species-level host identification of clinically relevant antibiotic resistance genes in environmental microbial communities. *Environ. Int.* **197**, 109337 (2025). 39978216 10.1016/j.envint.2025.109337

[9] Kent, A. G., Vill, A. C., Shi, Q., Satlin, M. J. & Brito, I. L. Widespread transfer of mobile antibiotic resistance genes within individual gut microbiomes revealed through bacterial Hi-C. *Nat. Commun.* **11**, 4379 (2020). 32873785 10.1038/s41467-020-18164-7 PMC7463002

[10] Zou, Y. et al. Deciphering the extracellular and intracellular antibiotic resistance genes in multiple environments reveals the persistence of extracellular ones. *J. Hazard. Mater.* **429**, 128275 (2022). 35093750 10.1016/j.jhazmat.2022.128275

[11] Peek, J. et al. Environmental resistome–guided development of resistance-tolerant antibiotics. *Proc. Natl. Acad. Sci. USA* **122**, e2504781122 (2025). 40388614 10.1073/pnas.2504781122 PMC12130845

[12] Böhm, M.-E., Razavi, M., Marathe, N. P., Flach, C.-F. & Larsson, D. G. J. Discovery of a novel integron-borne aminoglycoside resistance gene present in clinical pathogens by screening environmental bacterial communities. *Microbiome* **8**, 10.1186/s40168-020-00814-z (2020). 10.1186/s40168-020-00814-z PMC7085159 32197644

[13] Pei, Y. et al. ARGNet: using deep neural networks for robust identification and classification of antibiotic resistance genes from sequences. *Microbiome* **12**, 84 (2024). 38725076 10.1186/s40168-024-01805-0 PMC11080312

[14] Pereira, M. B., Wallroth, M., Jonsson, V. & Kristiansson, E. Comparison of normalization methods for the analysis of metagenomic gene abundance data. *BMC Genom.* **19**, 274 (2018). 10.1186/s12864-018-4637-6 PMC5910605 29678163

[15] Jonsson, V., Österlund, T., Nerman, O. & Kristiansson, E. Modelling of zero-inflation improves inference of metagenomic gene count data. *Stat. Methods Med. Res.* **28**, 3712–3728 (2019). 30474490 10.1177/0962280218811354

[16] Larsson, D. G. J., Flach, C.-F. & Laxminarayan, R. Sewage surveillance of antibiotic resistance holds both opportunities and challenges. *Nat. Rev. Microbiol.* **21**, 213–214 (2022). 36470999 10.1038/s41579-022-00835-5 PMC9734844

